# Weight Change Is a Characteristic Non-Motor Symptom in Drug-Naïve Parkinson’s Disease Patients with Non-Tremor Dominant Subtype: A Nation-Wide Observational Study

**DOI:** 10.1371/journal.pone.0162254

**Published:** 2016-09-13

**Authors:** Jun Kyu Mun, Jinyoung Youn, Jin Whan Cho, Eung-Seok Oh, Ji Sun Kim, Suyeon Park, Wooyoung Jang, Jin Se Park, Seong-Beom Koh, Jae Hyeok Lee, Hee Kyung Park, Han-Joon Kim, Beom S. Jeon, Hae-Won Shin, Sun-Ah Choi, Sang Jin Kim, Seong-Min Choi, Ji-Yun Park, Ji Young Kim, Sun Ju Chung, Chong Sik Lee, Tae-Beom Ahn, Won Chan Kim, Hyun Sook Kim, Sang Myung Cheon, Jae Woo Kim, Hee-Tae Kim, Jee-Young Lee, Ji Sun Kim, Eun-Joo Kim, Jong-Min Kim, Kwang Soo Lee, Joong-Seok Kim, Min-Jeong Kim, Jong Sam Baik, Ki-Jong Park, Hee Jin Kim, Mee Young Park, Ji Hoon Kang, Sook Kun Song, Yong Duk Kim, Ji Young Yun, Ho-Won Lee, In-Uk Song, Young H. Sohn, Phil Hyu Lee, Jeong-Ho Park, Hyung Geun Oh, Kun Woo Park, Do-Young Kwon

**Affiliations:** 1 Department of Neurology, Samsung Medical Center, Sungkyunkwan University School of Medicine, Seoul, Republic of Korea; 2 Neuroscience Center, Samsung Medical Center, Seoul, Republic of Korea; 3 Department of Neurology, Chungnam National University Hospital, Chungnam National University School of Medicine, Daejeon, Republic of Korea; 4 Department of Neurology, Soonchunhyang University Seoul Hospital, Soonchunhyang University College of Medicine, Seoul, Republic of Korea; 5 Department of Biostatistics, Soonchunhyang University Seoul Hospital, Soonchunhyang University College of Medicine, Seoul, Republic of Korea; 6 Department of Neurology, Gangneung Asan Hospital, College of Medicine, University of Ulsan, Gangneung, Republic of Korea; 7 Department of Neurology, Haeundae Paik Hospital, Inje University College of Medicine, Busan, Republic of Korea; 8 Department of Neurology, Korea University Guro Hospital, Korea University College of Medicine, Seoul, Republic of Korea; 9 Department of Neurology, Pusan National University Yangsan Hospital, Yangsan, Republic of Korea; 10 Department of Neurology, College of Medicine, Inje University, Ilsan-Paik Hospital, Ilsan, Republic of Korea; 11 Department of Neurology, College of Medicine, Seoul National University, Seoul, Republic of Korea; 12 Department of Neurology, Chung-Ang University College of Medicine, Seoul, Republic of Korea; 13 Department of Neurology, National Health, Insurance Corporation Ilsan Hospital, Ilsan, Republic of Korea; 14 Department of Neurology, Inje University, Busan Paik Hospital, Busan, Republic of Korea; 15 Department of Neurology, Chonnam National University Medical School, Gwangju, Republic of Korea; 16 Department of Neurology, Presbyterian Medical Center, Jeonju, Republic of Korea; 17 Department of Neurology, College of Medicine, Inje University Seoul Paik Hospital, Seoul, Republic of Korea; 18 Department of Neurology, College of Medicine, University of Ulsan, Seoul, Republic of Korea; 19 Department of Neurology, Kyung Hee University College of Medicine, Seoul, Republic of Korea; 20 Department of Neurology, CHA University College of Medicine, Bundang, Republic of Korea; 21 Department of Neurology, Dong-A University College of Medicine, Busan, Republic of Korea; 22 Department of Neurology, College of Medicine, Hanyang University, Seoul, Republic of Korea; 23 Department of Neurology, Seoul National University Boramae Hospital, Seoul, Republic of Korea; 24 Department of Neurology, College of Medicine, Chungbuk National University Hospital, Jeonju, Republic of Korea; 25 Department of Neurology, Pusan National University School of Medicine and Medical Research Institute, Busan, Republic of Korea; 26 Department of Neurology, Seoul National University Bundang Hospital, Bundang, Republic of Korea; 27 Department of Neurology, College of Medicine, the Catholic University of Korea, Seoul, Republic of Korea; 28 Department of Neurology, Kosin University College of Medicine, Busan, Republic of Korea; 29 Department of Neurology, College of Medicine, Inje University, Sanggye Paik Hospital, Seoul, Republic of Korea; 30 Department of Neurology, Gyeongsang National University School of Medicine, Jinju, Republic of Korea; 31 Department of Neurology, Konkuk University School of Medicine, Seoul, Republic of Korea; 32 Department of Neurology, Yeungnam University College of Medicine, Daegu, Republic of Korea; 33 Department of Neurology, Jeju National University Hospital, Jeju, Republic of Korea; 34 Department of Neurology, Konyang University Hospital, Daejeon, Republic of Korea; 35 Department of Neurology, Ewha Womans University Mokdong Hospital, Seoul, Republic of Korea; 36 Department of Neurology, School of Medicine, Kyungpook National University, Daegu, Republic of Korea; 37 Department of Neurology, College of Medicine, the Catholic University of Korea, Incheon, Republic of Korea; 38 Department of Neurology, Yonsei University, College of Medicine, Seoul, Republic of Korea; 39 Department of Neurology, Soonchunhyang University Bucheon Hospital, Soonchunhyang University College of Medicine, Bucheon, Republic of Korea; 40 Department of Neurology, Soonchunhyang University Cheonan Hospital, Soonchunhyang University College of Medicine, Cheonan, Republic of Korea; 41 Department of Neurology, Korea University Anam Hospital, Korea University College of Medicine, Seoul, Republic of Korea; 42 Department of Neurology, Korea University Ansan Hospital, Korea University College of Medicine, Ansan, Republic of Korea; Oslo Universitetssykehus, NORWAY

## Abstract

Despite the clinical impact of non-motor symptoms (NMS) in Parkinson’s disease (PD), the characteristic NMS in relation to the motor subtypes of PD is not well elucidated. In this study, we enrolled drug-naïve PD patients and compared NMS between PD subtypes. We enrolled 136 drug-naïve, early PD patients and 50 normal controls. All the enrolled PD patients were divided into tremor dominant (TD) and non-tremor dominant (NTD) subtypes. The Non-Motor Symptom Scale and scales for each NMS were completed. We compared NMS and the relationship of NMS with quality of life between normal controls and PD patients, and between the PD subtypes. Comparing with normal controls, PD patients complained of more NMS, especially mood/cognitive symptoms, gastrointestinal symptoms, unexplained pain, weight change, and change in taste or smell. Between the PD subtypes, the NTD subtype showed higher total NMS scale score and sub-score about weight change. Weight change was the characteristic NMS related to NTD subtype even after controlled other variables with logistic regression analysis. Even from the early stage, PD patients suffer from various NMS regardless of dopaminergic medication. Among the various NMS, weight change is the characteristic NMS associated with NTD subtype in PD patients.

## Introduction

From early stage, Parkinson’s disease (PD) patients suffer from various non-motor symptoms (NMS) as well as classic motor symptoms, and NMS can impact the quality of life even more than motor symptoms as the disease progresses [1–3]. Usually subtypes of PD are determined by main motor symptoms [4], and it is well-known that PD patients with non-tremor dominant (NTD) subtype show more severe motor symptoms and aggressive disease progression than those with tremor-dominant (TD) subtype [5, 6]. Similarly, NMS could be regarded to be more severe in PD patients with NTD subtype than those with TD subtype. Although previous studies already reported the difference in some NMS among PD subtypes, but these previous studies focused only few specific NMS among PD subtypes, not the whole spectrum of non-motor involvement [7–10]. Recently, there was only one study with systematic assessment of whole spectrum of NMS among PD subtypes, but this study investigated only the number of involved NMS among the PD subtypes, not the severity of NMS [11].

Furthermore, most of previous studies enrolled PD patients irrespective of PD medication, and failed to eliminate the confounding effects from medications [7–11]. Considering that dopaminergic medications have various effects on NMS in PD patients [12], confounding effects from medications should be considered during the investigation of motor or NMS in PD patients. Only few studies enrolled drug-naïve PD patients, but these studies mainly focused on premotor NMS, not the whole pattern of non-motor involvement in relation to the PD subtypes [13, 14].

In this study, to elucidate the characteristic non-motor features of each PD subtype, we investigated the whole pattern of NMS in drug-naïve PD patients, and compared between subtypes.

## Methods

### Subjects

This study was approved by the Institutional Review Board of each involved hospital, and all enrolled subjects provided written informed consent. We conducted nation-wide observational study to compare NMS between PD subtypes in drug-naïve PD patients.

We enrolled drug-naïve, early PD patients (disease duration of less than 4 years) and age- and sex-matched normal controls at movement disorders clinics in 40 community-based hospitals in Korea from May 2012 to December 2012. All PD patients were diagnosed with the United Kingdom Parkinson’s Disease Society Brain Bank criteria [15], and divided into two subgroups based on the PD subtype for the main motor symptoms: tremor dominant (TD) subtype and non-tremor dominant (NTD) subtype (indeterminate subtype and postural instability and gait disturbance subtype) [4].

Subjects with structural brain lesions, other known neurodegenerative disease, cognitive impairment (mini-mental state examination score of < 20 or fulfillment of *DSM-IV* criteria for dementia), psychiatric disorders needing medication, malignancy, or joint problems mimicking parkinsonism were excluded.

### Clinical assessment

Demographic and clinical data, including scales, were collected by interview and examination with movement disorder specialists at each hospital. Motor symptoms were evaluated with the Unified Parkinson Disease Rating Scale (UPDRS) part 3 and the Hoehn and Yahr (HY) scale [16]. Whole pattern of non-motor involvements were assessed by the Non-Motor Symptom Assessment Scale (NMSS) for Parkinson’s disease [17], and each NMS was also assessed with symptom-specific scales, including the Korean version of the Montreal Cognitive Assessment (MoCA-K) and Frontal Assessment Battery for cognitive evaluation [18, 19]; the Neuropsychiatric Inventory, Beck Depression Inventory (BDI) and Beck’s Anxiety Inventory (BAI) for neuropsychiatric assessment [20–22]; and the PD sleep scale (PDSS) and Parkinson Fatigue Scale (PFS) for sleep and fatigue evaluation [23, 24].

### Statistical analysis

All data were presented as mean and standard deviation. Demographic and clinical data were compared between groups (normal controls and PD patients; TD and non-TD subtypes) by using independent t-test or Mann-Whitney U-test for continuous variables, and Pearson’s χ^2^ or Fisher’s exact test for categorical variables. To investigate characteristic NMS related to PD subtype, multivariate analysis was carried out with logistic regression model with demographic and clinical variables including motor and NMS. The results of logistic regression were presented using 95% confidence intervals (CIs). *p*-values < 0.05 were considered statistically significant. Statistical analyses were performed with a commercially available software package (PASW version 18.0; SPSS Inc., Chicago, IL, USA).

## Results

### Comparison of non-motor symptoms between PD patients and normal controls

We enrolled 136 drug-naïve patients with early stage PD and 50 normal control subjects. Demographic and clinical data of all subjects were shown in Table 1. Compared with normal controls, PD patients reported higher total NMSS score, and subscores of mood/cognition, gastrointestinal tract, unexplained pain, change in ability to taste or smell, and weight change (Table 2). Similarly, PD patients also complained of more mood/cognitive symptoms (higher scores on the BAI, and lower MoCA-K scores) than normal controls.

**Table 1:** Demographic characteristics of enrolled subjects.

	Parkinson’s disease patients			Normal Control (n = 50)	*p*^a^	*p*^b^
	Total (n = 136)	TD (n = 68)	NTD (n = 68)			
Age, years	65.1 ± 8.7	65.1 ± 8.6	65.2 ± 8.9	65.4 ± 8.0	0.941	0.963
Sex, male, n (%)	58 (42.6)	32 (47.1)	26 (38.2)	17 (34)	0.315	0.386
BMI, UD/NR/OW/OB, n	8/41/35/52	5/20/18/25	3/21/17/27			0.913
Education, years	10.5 ± 4.6	10.7 ± 4.7	9.4 ± 4.8	10.3 ± 3.4	0.829	0.225
Disease duration, month	14.8 ± 11.7	15.4 ± 11.6	14.2 ± 11.9			0.500
HY stage 1/2/3, n (%)	57/76/3 (41.9/55.9/2.2)	29/38/1 (42.6/55.9/1.5)	28/38/2 (41.2/55.9/2.9)			0.642
UPDRS part 3	16.6 ± 8.0	16.3 ± 8.7	16.7 ± 7.4	1.0 ± 2.1	<0.001	0.476

PD, Parkinson’s disease; BMI, body mass index; UD, underweight; NR, normal range; OW, overweight; OB, obese; HY, Hoehn and Yahr; UPDRS, Unified Parkinson’s Disease Rating Scale.

^a^ comparison between PD and normal control.

^b^ comparison between tremor dominant and non-tremor dominant subtypes.

**Table 2:** Comparisons of non-motor symptoms between Parkinson’s disease patients and normal controls.

	PD patients (n = 136)	Normal controls (n = 50)	*p*
**Non-Motor Symptoms Assessment Scale**			
Total score	19.9 ± 19.4	13.9 ± 19.7	0.008
Cardiovascular including falls	0.7 ± 1.5	0.8 ± 2.1	0.297
Sleep/fatigue	3.1 ± 4.5	2.6 ± 4.1	0.235
Mood/cognition	4.2 ± 6.7	1.8 ± 3.4	0.003
Perceptual problems/hallucinations	0.1 ± 0.9	0.3 ± 1.6	0.118
Attention/memory	1.9 ± 2.6	1.8 ± 2.7	0.389
Gastrointestinal tract	1.4 ± 2.8	0.9 ± 2.7	0.018
Urinary	3.8 ± 5.6	2.8 ± 4.5	0.107
Sexual function	2.3 ± 5.3	1.9 ± 5.3	0.141
Unexplained pain	0.7 ± 1.8	0.2 ± 0.8	0.006
Change in ability to taste or smell	1.0 ± 2.1	0.4 ± 1.1	0.003
Weight change (not related to diet)	0.3 ± 1.3	0.1 ± 0.6	0.035
Excessive sweat (not related to weather)	0.5 ± 1.8	0.3 ± 1.1	0.411
**Other Scales on Non-motor Symptoms**			
Parkinson’s Disease Sleep Scale	125.4 ± 23.8	122.4 ± 25.3	0.299
Parkinson Fatigue Scale	34.5 ± 17.3	29.9 ± 26.2	0.010
Beck Depression Inventory	7.8 ± 7.0	7.1 ± 6.7	0.230
Beck Anxiety Inventory	7.1 ± 7.5	5.7 ± 7.7	0.003
MoCA-K	24.3 ± 3.9	25.9 ± 3.1	0.010

PD, Parkinson’s disease; TD, tremor dominant subtype; NTD, non-tremor dominant subtype; MoCA-K, Korean version of the Montreal Cognitive Assessment.

### Comparison of non-motor symptoms between PD subtypes

Of 136 enrolled PD patients, 68 subjects were PD patients with the TD subtype and 68 were those with the NTD subtype. Although the demographic and clinical data did not differ significantly between two PD subtypes, PD patients with NTD subtype patients showed higher total NMSS score and the only subscore of weight change domain comparing with those with TD subtype (Table 3). In addition, there were no significant differences in PDSS, PFS, BDI, BAI, MoCA-K score.

**Table 3:** Comparison of non-motor symptoms between subtypes of drug naïve Parkinson’s disease.

	TD (n = 68)	NTD (n = 68)	*p*
**Non-motor Symptom Scale**			
Total score	17.0 ± 20.8	23.0 ± 17.7	0.003
Cardiovascular including falls	0.7 ± 1.7	0.7 ± 1.4	0.600
Sleep/fatigue	2.8 ± 4.6	3.3 ± 4.5	0.300
Mood/cognition	4.1 ± 8.0	4.4 ± 5.2	0.179
Perceptual problems/hallucinations	0.2 ± 1.2	0.1 ± 0.6	0.493
Attention/memory	1.4 ± 2.2	2.3 ± 2.8	0.057
Gastrointestinal tract	1.3 ± 2.8	1.6 ± 2.7	0.107
Urinary	3.3 ± 4.2	4.4 ± 6.7	0.384
Sexual function	1.7 ± 4.6	2.9 ± 6.0	0.189
Unexplained pain	0.5 ± 1.4	0.9 ± 2.2	0.246
Change in ability to taste or smell	0.7 ± 1.7	1.3 ± 2.5	0.059
Weight change (not related to diet)	0.1 ± 0.3	0.5 ± 1.8	0.031
Excessive sweat (not related to weather)	0.3 ± 1.1	0.7 ± 2.3	0.300
**Other Scales on Non-motor Symptoms**			
Parkinson’s Disease Sleep Scale	122.0 ± 27.7	128.8 ± 18.9	0.303
Parkinson Fatigue Scale	32.5 ± 16.7	36.3 ± 17.7	0.288
Beck Depression Inventory	6.9 ± 6.8	8.6 ± 7.2	0.122
Beck Anxiety Inventory	6.4 ± 6.8	7.8 ± 8.2	0.333
MoCA-K	24.4 ± 4.0	24.3 ± 3.9	0.873

TD, tremor dominant subtype; NTD, non-tremor dominant subtype; MoCA-K, Korean version of the Montreal Cognitive Assessment.

### Characteristic non-motor symptom associated with PD subtype

To elucidate the characteristic NMS in PD subtype, multivariate logistic regression analysis was done with PD subtype as the dependent variable. Included in the model were demographic and clinical data (age, sex, education year, disease duration), UPDRS part 3 score and subscores of each NMSS domain. The only predictor for NTD subtype was subscore of weight change domain in NMSS (Table 4).

**Table 4:** Multiple logistic regression analysis; predictors for PD patients with non-tremor dominant subtype.

Variables	OR (95% CI)	*p*
Age, per year increase	1.002 (0.953–1.054)	0.923
Sex (male as reference)	0.540 (0.223–1.311)	0.173
Education year, per year increase	0.981 (0.890–1.081)	0.698
Disease duration, per month increase	0.994 (0.692–1.027)	0.713
UPDRS part 3 score, per score increase	1.002 (0.951–1.055)	0.945
**NMSS domains, per score increase**		
Cardiovascular including falls	1.040 (0.777–1.392)	0.793
Sleep/fatigue	0.897 (0.777–1.036)	0.140
Mood/cognition	0.973 (0.900–1.052)	0.491
Perceptual problems/hallucinations	0.961 (0.583–1.583)	0.875
Attention/memory	1.158 (0.948–1.414)	0.150
Gastrointestinal tract	1.030 (0.885–1.199)	0.699
Urinary	1.052 (0.950–1.165)	0.332
Sexual function	1.046 (0.964–1.134)	0.282
Unexplained pain	1.159 (0.866–1.515)	0.282
Change in ability to taste or smell	1.055 (0.866–1.285)	0.594
Weight change (not related to diet)	3.682 (1.143–11.865)	0.029
Excessive sweat (not related to weather)	1.128 (0.850–1.497)	0.405

PD, Parkinson’s disease; NMSS, Non-motor symptoms assessment scale.

## Discussion

This is the first study with systematic investigation of non-motor involvement between PD motor subtypes in drug-naïve PD patients. In our study, with multivariate regression analysis we demonstrated weight change was the only characteristic NMS in PD patients with NTD, and suggested weight change should be concerned even from early stage of PD. Considering that PD is a broad-spectrum disorder with clinical heterogeneity, each NMS can be easily affected by other motor and non-motor parkinsonian symptoms. Therefore, it is important to evaluate whole pattern of non-motor involvement in PD patients. Moreover, we enrolled drug-naïve PD patients, and eliminated the confounding effect from dopaminergic medications. Additionally, we also minimized possible confounding effect from the interrelation among each motor and non-motor parkinsonian symptoms by logistic regression analysis.

It is well-known that NMS are widely involved in PD patients from an early stage and some of NMS can even precede parkinsonian motor symptoms [1, 14]. We also demonstrated various NMS in PD patients, including pre-motor symptoms. When we compared between two subtypes, there was more non-motor involvements in PD patients with NTD subtype than those with NTD, even from early stage of PD. Among the various NMS, weight change was the characteristic NMS related with NTD subtype in PD patients.

Most of previous studies demonstrated weight loss in PD patients [10, 25, 26], while one study reported weight gain in early stage of PD [27]. Weight loss is known to be prominent with disease progression, but can be shown from early stage, and even several years before diagnosis [10, 25, 26]. In accordance with previous studies, we also found more weight change in early stage of PD patients, compared with normal controls.

The weight loss in PD is regarded as heterogeneous and multi-factorial symptom, and the pathophysiology of weight loss in PD patients is not clearly elucidated yet. In previous studies, various clinical features were known to contribute to weight loss, including poor appetite, hyposmia, intestinal hypomotility, depression, gastrointestinal side effects from medications, parkinsonism, dyskinesia, dementia and hallucination [26, 28, 29]. Considering we enrolled drug-naïve, early stage PD patients, our results suggest that parkinsonism of early or pre-motor stage, such as hyposmia, poor appetite or gastrointestinal hypomotility, can be closely associated with weight loss rather than those of advanced stage or medication side effects. Pathologic involvements in olfactory nerve, salivary glands and gastrointestinal tracts were already reported in pre-motor stage of PD patients, and also support our suggestion [30].

When we compared NMS between two PD subtypes, PD patients with NTD showed higher total NMSS score. In terms of each NMSS domains, there was significant difference only in subscore of weight loss domain. Previous study showed marginal significance in presence of tremor between PD patients with and without weight loss, although this study did not rule out medication effects [10]. In our study, to eliminate possible confounding effects from various interactions between weight change and other clinical factors [26, 28, 29], logistic regression analysis was done, and weight change was still the characteristic NMS in PD patients with NTD subtype. Therefore, weight change is characteristic NMS in PD patients with NTD regardless of other motor and NMS.

Interestingly, weight loss is known to be related with dementia, hallucination, and falling, which are common in PD patients with NTD subtype [26]. As already mentioned earlier, we suggest weight loss is regraded to be associated with early parkinsonian symptoms rather than late symptoms. Thus, characteristic non-motor involvement of NTD subtype starts from weight change, and evolves into diverse symptoms including perceptual and cognitive problems with disease progression.

Considering more severe motor symptoms in NTD subtype [5], PD patients with the NTD subtype show more both motor and non-motor involvement. Previous studies reported PD patients with NTD subtype have more degeneration of non-dopaminergic system as well as dopaminergic system [31, 32]. Further, more pathologic involvements were shown in NTD subtype patients [6]. Our results also support more clinical progression in PD patients with NTD subtype compared with those with TD subtype, even from early stage. In accordance with our results, recent study also reported weight loss is related with more severe neurodegeneration [33].

This study has some limitations. We designed to investigate whole NMS in drug-naïve PD patients, and did not check objective parameters for weight change, such as body mass index. However, unlike previous studies focusing on weight loss itself, we checked other NMS and minimized the interactions between weight loss and other motor and NMS. Second, we enrolled early stage PD patients, and we could not find any difference in NMS which is usually common in advanced stage of PD, such as cognitive or perceptual problems. In our study, PD patients with NTD subtype reported higher score of BDI and BAI than those with TD subtype, but there was no statistical significance. Considering NMS were reported to be prominent even from early stage of PD [1, 14], we focused on the early feature of non-motor involvement between PD subtypes, and we were able to avoid possible confounding effects by drug-naïve patients. Further study with follow-up data could be helpful to cover all these NMS. Lastly, we recruited a small number of subjects, especially for the normal control group. However, the primary objective of this study was to elucidate the non-motor involvement related with PD subtype, and enrolled sufficient number of drug-naïve, early PD patients. Additionally, we precisely evaluated non-motor involvements using the NMSS and other NMS-specific scales, and even with the small sample size, we were able to identify differences in NMS between PD patients and normal controls, and between PD subtypes.

In conclusion, our study demonstrated more non-motor involvement in PD patients with NTD subtype, and weight change as the characteristic NMS related with NTD subtype. Based on our results, we suggest that NTD subtype is associated with more widespread and severe clinico-pathologic involvement related to both motor and NMS even at an early stage.
